# Spectroscopy in the Second Minimum: Isotopic Limits, Lifetimes, and Magnetic Properties of Superdeformed *Tl* Nuclei

**DOI:** 10.6028/jres.105.021

**Published:** 2000-02-01

**Authors:** W. Reviol

**Affiliations:** University of Tennessee, Knoxville, TN 37996-1200, USA

**Keywords:** lifetime measurements, superdeformed bands, thallium

## Abstract

In-beam γ-ray spectroscopic studies of superdeformed bands in the mass 190 region are discussed with emphasis on recent results for Tl nuclei. Among those results, superdeformation at the edge of the region (in ^189^Tl) and the first measurement of a superdeformed quadrupole moment in an odd-*Z* nucleus (^191^Tl) is presented. The experiments were conducted with the Gammasphere array at the Lawrence Berkeley National Laboratory. They are examples for the type of physics addressed in the first phase of Gammasphere operation.

## 1. Introduction

Several regions of nuclei which possess a superdeformed, second minimum in their potential energy surface have been discovered [1]. In most cases, this second minimum gives rise to high-spin superdeformed bands, typically populated with a percent of the total cross section or less. The region of superdeformation located around ^192^Hg [2] is characterized by a prolate shape with an axis ratio of about 1.65 : 1, as inferred from lifetime measurements, and rotational bands with an essentially constant γ-ray energy separation Δ*E*_γ_ ≈ 40 keV that slightly decreases with increasing spin. The most recent finding are two superdeformed bands in ^189^Tl [3] which represents now the lightest superdeformed nucleus (neutron number *N* = 108) in the mass 190 region. These data shed light on the integrity of the second minimum at the edge of the superdeformed island and provide an important test for model predictions (e.g. in Ref. [4]). Other topics of current interest in the mass 190 region are associated with a detailed spectroscopy in the superdeformed well, such as one-step discrete linking transitions between the superdeformed and the normal deformed well [5], precision lifetime measurements to study the superdeformed shape as a function of *N* and atomic number *Z*, or measurements of magnetic properties of superdeformed states in odd-mass nuclei to identify the active proton or neutron orbitals. The latter two aspects are a main focus in the present contribution and new results for ^191^Tl are presented.

Probably all recent spectroscopic studies of superdeformation have benefited greatly from the new-generation of large arrays of Compton-suppressed Germanium detectors, Gammasphere [6,7], Eurogam [8,9], and others. The measurements presented in this contribution required a detector array with the “resolving power” of Gammasphere—i.e., its ability to resolve and identify weak γ-ray cascades from a large number of cascades through high-fold coincidence measurements (see, e.g., Ref. [7]). They were carried out with Gammasphere during its stay at the Lawrence Berkeley National Laboratory.

## 2. Experimental Details

Sets of experiments on both ^189^Tl and ^191^Tl were performed at the 88-inch cyclotron at Berkeley. The nuclei of interest were populated in (HI,xn) reactions. The pertinent information about the experiments is summarized in Table 1. Only prompt γ-ray coincidences of fold 4 and higher were collected with the (fully implemented) Gammasphere array. Notice that an earlier experiment on ^189^Tl in the commissioning phase of the array (with 35 detectors) has been included for the purpose of a comparison.

**Fig. 1:**
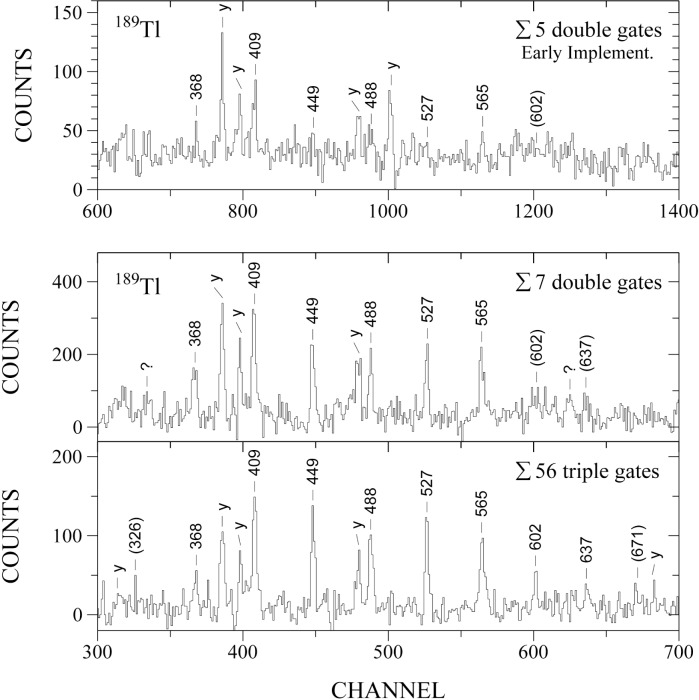
Comparison of γ-ray spectra of a superdeformed band in ^189^Tl obtained with the Early Implementation (top) and the Full Implementation (bottom) of the Gammasphere array. The band represents at most 0.2 % of the reaction yield. Transitions of the superdeformed band are labeled in keV, prominent transitions between normally deformed states near the yrast line are marked by “y”. The two bottom spectra are from the same data, applying double-gates on 3-fold concidences and triple-gates on 4-fold concidences, respectively.

Each of the recently observed superdeformed bands in ^189^Tl represents 0.1 % to 0.2 % of the total reaction yield [3]. Their observation provides a good example for the “resolving power” of the Gammasphere array. This feature of the array is emphasized in Fig. 1 where spectra for one of the superdeformed bands are shown as obtained from separate data and for different gating conditions. The top spectrum is the result of an experiment with the so-called Early Implementation of Gammasphere (35 detectors), obtained from double-gated 3-fold data. The members of the band ranging from 368 keV to 565 keV are eventually observed. However, in this spectrum the peak-to-background ratio is rather low and the strongest transitions in ^189^Tl (near the yrast-line) are still dominant. The advance comes with the fully implemented Gammasphere array, as indicated by the two bottom spectra. Its “resolving power” is about a factor of 10 higher than that of the Early Implementation version (and a factor of 100 higher than that of a previous generation array). Accordingly, about a factor of 10 is gained in the peak-to-background ratio compared to the top spectrum. Further improvement in the spectral quality is seen when applying combinations of triple coincidence gates (bottom most spectrum) rather than double gates. Additional transitions higher in the cascade are then observed that represent about one tenth of the intensity of the strongest inband transitions. That is, the sensitivity limit reached is about 10^−4^. By comparison, with the previous generation of detector arrays transitions with intensities down to 1 % of the reaction yield were observable.

## 3. Limits of the Island of Superdeformation

The band shown in Fig. 1 as well as the other superdeformed band in ^189^Tl [3] exhibit the characteristic spacing Δ*E*_γ_ ≈ 40 keV. Compared with the superdeformed bands reported for the heavier Tl isotopes [10–12], there is no significant difference in the moments of inertia (ℑ^(2)^ = 4/Δ*E*_γ_) as function of *N*. This is supportive for an essentially constant quadrupole deformation from the center of the island to its edges. However, the two superdeformed sequences in ^189^Tl are “shorter” than those in the heavier Tl isotopes. When one fits the moments of inertia in a Harris parametrization (see, e.g., Ref. [13]), one obtains values of 25/2 and 27/2 ℏ, respectively, for the spins at the bottom of the bands in ^189^Tl, as compared to values of 23/2 ℏ and lower for the bands in the heavier isotopes. Both the weak intensity of the superdeformed bands in ^189^Tl and their decay-out as a function of spin agree with the predicted isotopic trends and reflect a shallow second potential well at the low-*N* edge of the island of superdeformation.

While the isotopic boundary of the island seems to be established for the lightest isotopes (*N ≥* 108), its extension towards larger *N* is not clear yet. So far, the island has been established up to *N* = 116 (^198^Pb [1]). This limit, however, is based on a technical shortcoming—there are no suitable beam-target combinations to populate the next heavier isotopes at sufficiently high spin (> 40ℏ).

## 4. Detailed Spectroscopy in the Second Minimum

The superdeformed character of the most prominent bands in the mass 190 region (≈ 2 % of the reaction yield) was established in lifetime measurements using the Doppler Shift Attenuation Method (DSAM). While for this purpose a previous generation array was sufficient, a new generation array is necessary to obtain high quality DSAM data also for the much more weakly populated superdeformed bands (e.g., excited bands in the superdeformed well). The relative precision of the transition quadrupole moments derived from the measured lifetimes can be 5 % or better, if systematic errors are neglected. However, as DSAM relates decay time to the stopping time in a target backing, the large systematic uncertainties (≈ 10 % to 15 %) due to the problem of stopping power formulations still remain. Therefore, to be sensitive to small deformation differences between bands, several authors have carried out so-called Differential DSAM measurements (see, e.g., Refs. [14,15]). The basic idea for this type of measurement is to compare recoiling nuclei preferably of the same atomic number, made in the same reaction and recoiling in the same backing material, so that the systematic uncertain ties asscociated with the stopping process cancel out.

**Fig. 2:**
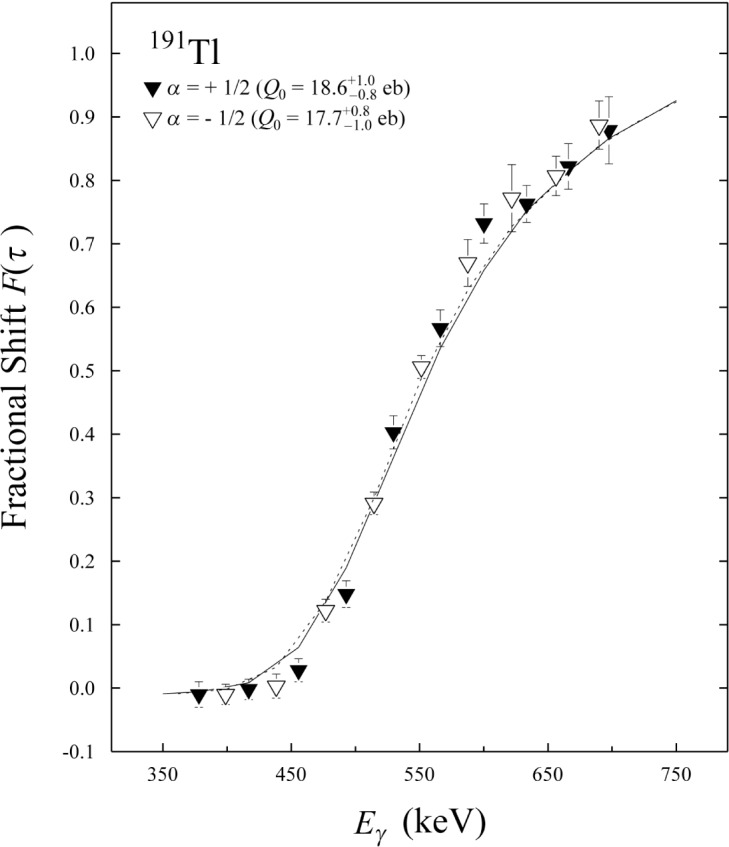
Fractional Doppler shifts *F*(*τ*) measured for γ rays in the two superdeformed bands of ^191^Tl versus transition energies. The solid and dashed curves represent the best fits corresponding to the *Q*_0_ values obtained for both bands (bands are labelled by their signature *α* [10]).

In the course of this work, a DSAM lifetime measurement has been carried out for the two superdeformed bands in ^191^Tl (cf. Table 1). These bands are viewed as signature partners [10] where each sequence represents about 0.5 % of the reaction yield. As is customary for weak γ-ray sequences, a centroid shift analysis has been performed. A triple-gating procedure was essential because of the many overlapping peaks. From the peak centroids measured at five different angle groups of the Gammasphere array, fractions of the full Doppler shift were determined according to the first-order relation $F\left(\tau \right)=\left(\langle E_{\gamma }\rangle -E_{\gamma }^{0}\right)/E_{\gamma }^{0}\beta_{0}\cos\Theta$. $E_{\gamma }^{0}$ is the nominal (unshifted) γ-ray energy, and 〈*E*_γ_〉 the corresponding energy measured at an average angle *Θ* representative for the group of detectors. The factor *β*_0_ refers to the intial velocity of the recoiling nucleus formed in the center of the target and was calculated to be *β*_0_ = 0.0182 *c*. In Fig. 2, these *F*(*τ*) values are presented as a function of γ-ray energy for the transitions in both bands.

From the experimental *F*(*τ*) values, one can extract a constant quadrupole moment *Q*_0_ for all levels within a band. The decay probability for each band member can be described by a rotational model with *Q*_0_ as free parameter. Besides, the sidefeeding into each state needs to be modelled (unless one is able to gate on top of the band). In order to extract intrinsic quadrupole moments *Q*_0_ for the two superdeformed bands in ^191^Tl from the present data, the code FITFTAU [15] has been used. In this fitting procedure, a constant sidefeeding quadrupole moment *Q*_SF_ (representing feeder cascades into each superdeformed state) and a “single” lifetime *T*_SF_ (one-step delay at the top of a feeder cascade) have been used as additional free parameters. The slowing-down times in both the target and the Au backing have been calculated with stopping powers provided by the code TRIM [16]. The results of these fits to the *F*(*τ*) data are illustrated by the curves plotted in Fig. 2. The quoted errors for the *Q*_0_ values include the covariance between all three fit parameters, but not the systematic uncertainties associated with the stopping powers.

The quadrupole moments obtained for both bands, $Q_{0}=18.6_{-0.8}^{+1.0},\;17.7_{-1.0}^{+0.8}$ eb (*B*(E2) values of 1700 W.u. to 2500 W.u.) are—as expected—very similar and correspond indeed to a superdeformed rotor in this mass region. This is the first measurement of the quadrupole moment in a superdeformed band of an odd-*Z* nucleus in the mass 190 region. Within the statistical uncertainties, the *Q*_0_ values obtained for ^191^Tl agree with the quadrupole moments measured in the neigboring nuclei ^192,194^Hg [15] and ^190^Hg [17]. For the purpose of a comparison between the Tl and Hg isotopes, one has to ignore the uncertainties in these data associated with different atomic numbers and recoil velocities. However, these discrepancies are rather minor since similar reactions have been used. It is save to conclude that the superdeformed Hg “core” is not much polarized by the additional proton. Interestingly, there is no measurable deformation change between the ^190,192,194^Hg isotopes either. The superdeformed shapes in the mass 190 region seem to be remarkably constant with respect to variations of *N* and *Z*.

Another advance that came with the new-generation arrays and should be briefly mentioned is the observation of magnetic properties in some superdeformed nuclei in the mass 190 region. This observation in the mass 190 region is eased by the fact that the superdeformed bands there occur at considerably lower spins (down to ≈ 10ℏ) and smaller transition energies than comparable bands in, e.g., the mass 150 region (> 20ℏ). For some of these superdeformed bands, a decay by magnetic dipole transitions, M1’s ( $T\propto E_{\gamma }^{3}$), becomes nearly competitive with the E2 transitions ( $T\propto E_{\gamma }^{5}$) at the bottom of the sequence. These are the signature partner superdeformed bands in odd-*Z* and odd-*N* nuclei, for which cross-talk between the E2 sequences and M1 interband transitions have been reported (see, e.g., Ref. [12]). The magnetic properties can then be inferred from *B*(M1)/*B*(E2) branching ratios—even though these interband transitions carry at most one tenth of the decay intensity of a competing E2 inband transition—and used to identify active orbitals in the superdeformed nucleus. With this procedure, one is sensitive only for the orbitals with medium to high K. On the other hand, a low-K configuration is ruled out by noticeable M1 strength.

The analysis of the magnetic properties of the signature partner bands in ^191^Tl strongly supports the prediction that the 81^st^ proton occupies the *i*_13/2_[642]5/2 Nilsson level [10]. The same proton *i*_13/2_ configuration assignment has been reported for the signature partner superdeformed bands in ^193,195^Tl [11,12]. However, with the additional *B*(E2) lifetime information for the bands in ^191^Tl the present result is obtained with a minimum of assumptions.

## 5. Conclusions

Measurements of transition quadrupole moments (*B*(E2)’s) and magnetic dipole moments (*B*(M1)’s or *g*-factors) play a crucial role in nuclear structure studies. The physics insight often comes from such measurements of electromagnetic moments. With the “resolving power” of modern arrays of Germanium detectors, detailed spectroscopy in the second well has become a reality. Recent experimental studies in the mass 190 region using these arrays have resulted in a rather complete and coherent picture for a prominent superdeformed island. For example, excitation energies, spins and probable parities for superdeformed states could be determined in some cases, based on the observation of one-step discrete decay-out γ-rays. More specific for the results presented in this contribution, the superdeformed shape in this mass region is found to be very stiff with respect to neutron number, proton number and orbital occupation, based on precise DSAM measurements in a series of nuclei. This is a remarkable feature in view of the variations in *Q*_0_ found in the mass 150 region of superdeformation. A consistent picture for both proton and neutron excitations in the superdeformed well has been obtained from the center of the mass 190 island to its edges. The configuration assignments rest to a significant amount on measured magnetic properties of superdeformed states, a rather unique feature of the mass 190 region.

## Figures and Tables

**Table 1:** Experimental conditions employed in this work

Reaction	*E*_beam_	Target foil	No. detectors	γ-fold	No. events
^156^Gd(^37^Cl,4n)^189^Tl	171 MeV	self-supporting	35^a^	≥3	3 × 10^8^
^156^Gd(^37^Cl,4n)^189^Tl	172 MeV	self-supporting	101	≥4	7 × 10^8^
^159^Tb(^36^S,4n)^191^Tl	165 MeV	with Au backing^b^	93	≥4	2 × 10^9^
^156^Gd(^37^Cl,4n)^191^Tl	132 MeV	self-supporting	93	≥4	1 × 10^9^

a Early implementation of Gammasphere.

b DSAM lifetime measurement (see Sec. 4).
